# Characterization of the pathogenicity of extraintestinal pathogenic *Escherichia coli* isolates from pneumonia-infected lung samples of dogs and cats in South Korea

**DOI:** 10.1038/s41598-023-32287-z

**Published:** 2023-04-05

**Authors:** Chi Sun Yun, Bo-Youn Moon, Mi-Hye Hwang, Soo-Kyoung Lee, Bok-Kyung Ku, Kichan Lee

**Affiliations:** Animal Disease Diagnostic Division, Animal and Plant Quarantine Agency, Gimcheon-si, South Korea

**Keywords:** Microbiology, Diseases, Pathogenesis

## Abstract

This study aimed to investigate the pathogenicity of extraintestinal pathogenic *Escherichia coli* (ExPEC) isolated from dog and cat lung samples in South Korea. A total of 101 *E. coli* isolates were analyzed for virulence factors, phylogroups, and O-serogroups, and their correlation with bacterial pneumonia-induced mortality was elucidated. P fimbriae structural subunit (*papA*), hemolysin D (*hlyD*), and cytotoxic necrotizing factor 1 (*cnf1*) were highly prevalent in both species, indicating correlation with bacterial pneumonia. Phylogroups B1 and B2 were the most prevalent phylogroups (36.6% and 32.7%, respectively) and associated with high bacterial pneumonia-induced mortality rates. Isolates from both species belonging to phylogroup B2 showed high frequency of *papA*, *hlyD*, and *cnf1*. O-serogrouping revealed 21 and 15 serogroups in dogs and cats, respectively. In dogs, O88 was the most prevalent serogroup (n = 8), and the frequency of virulence factors was high for O4 and O6. In cats, O4 was the most prevalent serogroup (n = 6), and the frequency of virulence factors was high for O4 and O6. O4 and O6 serogroups were mainly grouped under phylogroup B2 and associated with high bacterial pneumonia-induced mortality. This study characterized the pathogenicity of ExPEC and described the probability of ExPEC pneumonia-induced mortality.

## Introduction

*Escherichia coli* is the most common gram-negative bacterium in the gastrointestinal tract and causes extraintestinal infections in humans and animals, including urinary tract infections (UTIs), neonatal meningitis, prostatitis, and pneumonia^1–3^. *E. coli* strains that cause diseases other than those of the digestive organs are referred to as extraintestinal pathogenic *E. coli* (ExPEC)^4,5^. The pathogenicity of ExPEC is phylogenetically and epidemiologically different from that of commensal and intestinal *E. coli* strains. Previous reports showed that ExPEC strains contain specific virulence factor encoding genes: cytotoxic necrotizing factor 1 (*cnf1*), hemolysin D (*hlyD*), P fimbriae structural subunit (*papA*), S and F1C fimbriae subunits (*sfa*/*foc*), A firmbrial adhesins (*afa*), group 2 capsular polysaccharide units (*kps MII*), and aerobactin receptor (*iutA*)^1,6^.

Previous studies established the pathogenesis of ExPEC-induced UTIs^7,8^, meningitis^9^, sepsis^10,11^, and pyometra^12^ in various species. However, limited cases and analyses of ExPEC-induced pneumonia have been reported worldwide. *E. coli* isolates from the lungs of cats contained various virulence factor encoding genes, including *cnf1*, *papA*, *hlyD*, and *kps MII*^6,13^. In addition, *E. coli* isolates from dogs with pneumonia expressed *cnf1*, *papA*, *kps MII*, and *sfaS*^14,15^. We previously reported that *E. coli* isolated from the lungs of a dog with acute pneumonia in South Korea contained CNF1s and hemolysins. This was categorized as a case of ExPEC pneumonia-induced mortality in the absence of other infections^16^. However, there are limited number of ExPEC cases to establish the correlation between pneumonia and ExPEC, particularly the correlation between the frequency of occurrence of virulence factors and bacterial pneumonia-induced mortality.

This study aimed to investigate the pathogenicity and characteristics of ExPEC isolated from dog and cat lung samples in South Korea. The results established the correlation between bacterial pneumonia and various characteristics of ExPEC.

## Results

### Detection of virulence factors in *E. coli* isolates

PCR analysis of the 101 *E. coli* isolates from dog and cat lung samples revealed the presence of virulence factors (Table 1). The virulence factor *hlyD* was prevalent in the *E. coli* isolates from both dogs and cats (30.3% and 77.1%, respectively). The overall frequency of occurrence of *cnf1* in both species was 36.6%, with a relatively lower species-specific frequency of occurrence of 18.2% in dogs and 71.4% in cats. The virulence factor *iutA* was detected at a low frequency of 14.3% in cats and 34.8% in dogs. Other factors like *papA*, *kps MII*, and *focG* exhibited an overall frequency of occurrence of 33.7%, 30.7%, and 21.8%, respectively. The overall frequency of occurrence of *sfa* was 8.9% (species-specific frequency, 4.5% in dogs and 17.1% in cats). Virulence factors *stx1*, *stx2*, *eae*, and *afa* were not detected in any isolate (Table 2). Histopathological examination revealed that bacterial pneumonia-induced mortality was 28.7% (species-specific mortality rate, 25.8% in dogs and 34.3% in cats) (Table 2). The frequency of occurrence was high in *papA* (15/29 cases), *hlyD* (18/29 cases), and *cnf1* (15/29 cases), and low in *iutA* (4/29 cases) according to bacterial pneumonia-induced mortality, although there was no significant differences (*P* > 0.05). The frequency of occurrence of virulence factors *focG*, *kps MII*, *papA*, *hlyD* and *cnf1* was significantly higher in cats than in dogs (*P* < 0.05). *E. coli* isolates from lung samples of cats showed no significant differences in virulence factor distribution between cases of bacterial pneumonia-induced mortality and mortality due to other causes. However, in dogs, the distribution of *papA* (7/17 cases, 41.18%), *hlyD* (10/17 cases, 58.82%) and *cnf1* (7/17 cases, 41.18%) was significantly higher in *E. coli* isolates from cases of bacterial pneumonia-induced mortality than mortality due to other causes (*P* < 0.05).

**Table 1:** Primer sequences for identification of virulence factors.

Genes	Primer designation	Sequence 5ʹ → 3ʹ	Molecular weight (bp)	References
*focG*	focG_106F	cgtacctgtaccattggtaatggagg	366	Franz et al.^17^
	R focG_471R	tgaattaatacttcccgcaccagc		
*kpsMII*	kpsMII_121F	gcgcatttgctgatactgttg	452	
	kpsMII_572	gggaacatgatgcaggagatg		
*papA*	papA_67A	atggcagtggtgtcttttggtg	717	
	papA_+202R	cgtcccaccatacgtgctcttc		
*sfaS*	sfaS_210F	gtctctcaccggatgccagaatat	138	
	sfaS_347R	gcattacttccatccctgtcctg		
*afa*	afa F	ggcagagggccggcaacaggc	594	
	afa R	cccgtaacgcgccagcatctc		
*hlyD*	hlyD_92F	ctccggtacgtgaaaaggac	904	
	hlyD_995R	gccctgattactgaagcctg		
*iutA*	iutA 674F	atcggctggacatcatgggaac	314	
	iutA_987R	cgcatttaccgtcgggaacgg		
*stx1*	SLT6	accctgtaacgaagtttgcg	140	Clermont et al.^18^
	SLT7	atctcatgcgactacttgac		
*stx2*	SLT12	atcctattcccgggagtttacg	584	
	SLT13	gcgtcatcgtatacacaggagc		
*eae*	eae.f	gaacggcagaggttaatctg	203	
	eae.r	caatgaagacgttatagccc		
*cnf1*	CNF-1s	gggggaagtacagaagaatta	1111	Toth et al.^19^
	CNF-1as	ttgccgtccactctcaccagt

**Table 2:** Prevalence of virulence factors in *Escherichia coli* isolates from the lungs of dogs and cats.

Animal		No. of virulence factors (%)										
		*focG*	*kps MII*	*papA*	*sfaS*	*afa*	*hlyD*	*iutA*	*Stx1*	*Stx2*	*eae*	*cnf1*
Dogs (n = 66)	BPM (n = 17, 25.8%)	5	5	7^a^	1	0	10^a^	2	0	0	0	7^a^
	Non-BPM (n = 49, 74.2%)	4	9	5	2	0	10	21	0	0	0	5
	Total	9 (13.6)	14 (21.2)	12 (18.2)	3 (4.5)	0 (0.0)	20 (30.3)	23 (34.8)	0 (0.0)	0 (0.0)	0 (0.0)	12 (18.2)
Cats (n = 35)	BPM (n = 12, 34.3%)	3	5	8	2	0	8	2	0	0	0	8
	Non-BPM (n = 23, 65.7%)	10	12	14	4	0	19	3	0	0	0	17
	Total	13 (37.1)^b^	17 (48.6)^b^	22 (62.9)^b^	6 (17.1)	0 (0.0)	27 (77.1)^b^	5 (14.3)	0 (0.0)	0 (0.0)	0 (0.0)	25 (71.4)^b^
Total (n = 101)	BPM (n = 29, 28.7%)	8	10	15	3	0	18	4	0	0	0	15
	Non-BPM (n = 72, 71.3%)	14	21	19	6	0	29	24	0	0	0	22
	Total	22 (21.8)	31 (30.7)	34 (33.7)	9 (8.9)	0 (0.0)	47 (46.5)	28 (27.7)	0 (0.0)	0 (0.0)	0 (0.0)	37 (36.6)

*BPM* bacterial pneumonia-induced mortality.

^a^Significant difference (*P* < 0.05) between bacterial pneumonia-induced mortality and mortality due to other causes in dogs.

^b^Significant differences (*P* < 0.05) in the prevalence of *focG*, *kps MII*, *papA*, *hlyD* and *cnf1* between dogs and cats.

### Correlation between the phylogroups and virulence factors of *E. coli* isolates

Phylogenetic analysis revealed that 36.6% of *E. coli* isolates belonged to the phylogroup B2, 32.7% to group B1, 9.9% to group C, 8.9% to group A, 4.0% to group C, 3.0% to group F, 1.0% to group E, and unclassified isolates accounted for 4.0% of the total isolates (Table 3). Most isolates from cases of bacterial pneumonia-induced mortality were classified under phylogroups B1 and B2, while three cases were grouped under phylogroup C, D, and unclassified (one case in each group). In dogs, all phylogroups, including the unclassified group, were identified, and group B1 was the most prevalent (42.4%). Nine deaths due to bacterial pneumonia were reported in the B1 group, and *focG*, *hlyD*, and *iutA* were detected in these cases. The prevalence of phylogroup B2 in dogs was lower than the prevalence of group B1, but mortality due to bacterial pneumonia was detected more in phylogroup B2 (7/12 cases, *P* < 0.05), and all virulence factors apart from *iutA* were identified in this group. All isolates from dog lung samples, belonging to phylogroup B2 contained the virulence factor-encoding genes *papA*, *hlyD*, and *cnf1*. In isolates from dog lung samples belonging to phylogroups A, C, D, E, and F, mortality due to bacterial pneumonia was not established, and one case was grouped under the unclassified phylogroup. In isolates from cat lung samples, phylogroups B2 (71.4%), B1 (14.3%), C (11.4%), and D (2.9%) were identified. The most prevalent phylogroup was B2, and *hlyD* and *cnf1* were detected in most *E. coli* isolates from cat lung samples. Moreover, it was observed that in the *E. coli* isolates belonging to this B2 group, other virulence factors, including *papA*, *kps MII*, and *focG* were detected. In one of the two cases (from cat lung samples) of death due to bacterial pneumonia belonging to phylogroup B1, virulence factors *papA*, *sfaS*, *hlyD*, and *iutA* were detected, while the presence of *iutA* was confirmed in the other case. However, virulence factors were not identified in the mortality cases grouped under phylogroup C, and only *focG* was detected in one bacterial pneumonia-induced mortality case grouped under phylogroup D.

**Table 3:** Phylogroups and virulence factors in extraintestinal pathogenic *E. coli* isolates from the lung samples of dogs and cats.

Animal	Genes	No. of isolates belonging to phylogroup								
		A (n = 9, 13.6%)	B1 (n = 28. 42.4%)	B2 (n = 12, 18.1%)	C (n = 6, 9.0%)	D (n = 3, 4.5%)	E (n = 1, 1.5%)	F (n = 3, 4.5%)	Non-classified (n = 4, 6.0%)	Total (n = 66)
Dog	*focG*	1	4	2	–	–	–	–	2	9
	*kps MII*	–	–	9	–	2	–	1	2	14
	*papA*	–	–	12	–	–	–	–	–	12
	*sfaS*	–	–	3	–	–	–	–	–	3
	*hlyD*	1	4	12	–	2	–	–	1	20
	*iutA*	4	12	–	2	–	–	2	3	23
	*cnf1*	–	–	12	–	–	–	–	–	12
	Bacterial pneumonia-induced mortality	0	9	7^a^	0	0	0	0	1	17

		A (n = 0)	B1 (n = 5, 14.3%)	B2 (n = 25, 71.4%)	C (n = 4, 11.4%)	D (n = 1, 2.9%)	E (n = 0)	F (n = 0)	Non-classified (n = 0)	Total (n = 35)
Cat	*focG*	–	1	11	–	1	–	–	–	13
	*kps MII*	–	–	17	–	–	–	–	–	17
	*papA*	–	1	21	–	–	–	–	–	22
	*sfaS*	–	1	5	–	–	–	–	–	6
	*hlyD*	–	1	24	2	–	–	–	–	27
	*iutA*	–	2	2	1	–	–	–	–	5
	*cnf1*	–	1	24	–	–	–	–	–	25
	Bacterial pneumonia-induced mortality	–	2	8	1	1	–	–	–	12

		A (n = 9, 8.9%)	B1 (n = 33, 32.7%)	B2 (n = 37, 36.6%)	C (n = 10, 9.9%)	D (n = 4, 4.0%)	E (n = 1, 1.0%)	F (n = 3, 3.0%)	Non-classified (n = 4, 4.0%)	Total (n = 101)
Total	*focG*	1	5	13	–	1	–	–	2	22
	*kps MII*	–	–	26	–	2	–	1	2	31
	*papA*	–	1	33	–	–	–	–	–	34
	*sfaS*	–	1	8	–	–	–	–	–	9
	*hlyD*	1	5	36	2	2	–	–	1	47
	*iutA*	4	14	2	3	–	–	2	3	28
	*cnf1*	–	1	36	–	–	–	–	–	37
	Bacterial pneumonia-induced mortality	0	11	15	1	1	0	0	1	29

^a^Significant difference (*P* < 0.05) between different phylogroups based on bacterial pneumonia-induced mortality in dogs.

### Correlation between the O-serogroups and virulence factors of *E. coli* isolates

O-serogrouping of all isolates was performed and the frequency of occurrence of different virulence factors in the identified serogroups was evaluated (Table 4). In this study, 28 O-serogroups were identified, and 13 isolates contained unidentified serogroups (Table 4). Nine O-serogroups (O4, O6, O7, O8, O25, O29, O54, O88, and O128) were detected in both dogs and cats, with 13 groups identified from dog lung samples (O9, O11, O36, O41, O60, O78, O81, O89, O91, O131, O156, O161, and O166) and six from cat lung samples (O2, O22, O51, O56, O83, and O102). The most prevalent O-serogroup in dogs was O88 (n = 8), followed by O6 (n = 7), O8 (n = 6), O4 (n = 5), O89 (n = 4), O128 (n = 3), and O9 (n = 3). The detection of virulence factors was the highest in the O6 and O4 serogroups identified from dog lung sample isolates. Six isolates belonging to serogroup O6 were found to express *kps MII*, *papA*, *hlyD*, and *cnf1*, and all isolates grouped under serogroup O4 expressed *papA*, *hlyD*, and *cnf1*. Bacterial pneumonia-induced mortality was most prevalent in serogroup O4 (4 cases, *P* < 0.05), followed by O36 (3 cases), O54 (2 cases), and O6 (2 cases). In cat lung sample isolates, serogroup O4 was the most prevalent (6 cases), followed by O6 (5 cases), O8 (4 cases), O51 (4 cases) and O25 (3 cases), although there was no statistical association. All isolates belonging to serogroup O4 expressed *papA*, *hlyD*, and *cnf1*, and two cases of bacterial pneumonia-related death were reported. In all isolates belonging to serogroups O6, O25, and O51, apart from one case, *kps MII*, *papA*, *hlyD*, and *cnf1* were detected. In isolates grouped under serogroup O25, bacterial pneumonia-induced mortality was not reported. However, bacterial pneumonia-induced mortality was reported in isolates belonging to serogroups O6 (2 cases) and O51 (1 case). Only *hlyD* was detected in isolates from serogroup O8, and one case of bacterial pneumonia-induced death was confirmed in this serogroup. The correlation between phylogenetic groups and O-serogroups is shown in Table 5. Apart from phylogroup C of serogroup O8 (n = 4, dog lung samples and n = 3, cat lung samples) and phylogroup A of serogroup O89 (n = 3, dog lung samples), most *E. coli* isolates from dog and cat lung samples were classified under phylogroups B1 and B2, irrespective of the assigned O-serogroups.

**Table 4:** O-serogroups and virulence factors in extraintestinal pathogenic *E. coli* isolates from the lung samples of dogs and cats.

Animals	Genes	No. of isolates beloning to O-serogroup															
		O4 (n = 5)	O6 (n = 7)	O7 (n = 1)	O8 (n = 6)	O9 (n = 3)	O11 (n = 2)	O25 (n = 1)	O36 (n = 3)	O54 (n = 2)	O88 (n = 8)	O89 (n = 4)	O128 (n = 3)	O156 (n = 1)	O166 (n = 2)	Others (n = 9)*	Non (n = 10)
Dog	*focG*	1	1	–	–	–	–	–	2	1	–	–	–	–	–	–	5
	*kps MII*	3	6	1	–	–	–	1	–	–	–	–	–	–	2	1	1
	*papA*	5	6	–	–	–	–	–	1	–	–	–	–	–	–	–	–
	*sfaS*	0	3	–	–	–	–	–	–	–	–	–	–	–	–	–	–
	*hlyD*	5	6	1	1	1	–	–	1	–	–	–	–	1	2	1	3
	*iutA*	0	0	1	2	–	–	1	–	–	8	2	–	–	–	5	4
	*cnf1*	5	6	–	–	–	–	–	1	–	–	–	–	–	–	–	–
	BPM	4^a^	2	1	–	1	–	–	3	2	1	–	1	1	–	–	1

		O2 (n = 1)	O4 (n = 6)	O6 (n = 5)	O7 (n = 1)	O8 (n = 4)	O22 (n = 1)	O25 (n = 3)	O29 (n = 1)	O51 (n = 4)	O54 (n = 1)	O56 (n = 1)	O83 (n = 1)	O88 (n = 1)	O102 (n = 1)	O128 (n = 1)	Non (n = 3)
Cat	*focG*	1	4	–	1	–	1	3	–	–	–	1	–	–	–	1	1
	*kps MII*	1	–	5	–	–	1	3	–	4	–	1	1	–	–	–	1
	*papA*	1	6	5	–	–	–	2	–	4	–	1	1	1	–	–	1
	*sfaS*	–	1	–	–	–	–	3	–	–	–	1	–	1	–	–	-
	*hlyD*	1	6	5	–	2	1	3	–	4	1	1	1	1	–	–	1
	*iutA*	–	–	–	–	–	–	3	1	–	–	–	–	1	–	–	1
	*cnf1*	1	6	5	–	–	1	3	–	4	1	1	1	1	–	–	1
	BPM	–	2	3	–	1	–	–	1	1	–	–	–	1	–	1	2

*Non* non-typeable, *BPM* bacterial pneumonia-induced mortality.

*Others include O29, O41, O60, O78, O81, O91, O131 and O161.

^a^Significant difference (*P* < 0.05) between different O-serogroups based on bacterial pneumonia-induced mortality.

**Table 5:** Phylogroups and O-serogroups in extraintestinal pathogenic *E. coli* isolates from the lung samples of dogs and cats.

Animals	Phylogroups	No. of isolates belonging to O-serogroup and phylogroup															
		O4 (n = 5)	O6 (n = 7)	O7 (n = 1)	O8 (n = 6)	O9 (n = 3)	O11 (n = 2)	O25 (n = 1)	O = 36 (n = 3)	O54 (n = 2)	O88 (n = 8)	O89 (n = 4)	O128 (n = 3)	O156 (n = 1)	O166 (n = 2)	Others (n = 9)*	Non (n = 10)
Dog	A	–	–	–	–	1	–	–	–	–	–	4	–	–	–	2	2
	B1	–	1	–	2	2	2	–	2	2	8	–	3	1	–	2	5
	B2	5	6	–	–	–	–	–	1	–	–	–	–	–	–	–	–
	C	–	–	–	4	–	–	–	–	–	–	–	–	–	–	1	1
	D	–	–	–	–	–	–	–	–	–	–	–	–	–	2	1	–
	E	–	–	–	–	–	–	–	–	–	–	–	–	–	–	1	–
	F	–	–	–	–	–	–	1	–	–	–	–	–	–	–	1	1
	non	–	–	1	–	–	–	–	–	–	–	–	–	–	–	1	3
	BPM	B2 = 4	B2 = 2	non = 1	–	B1 = 1	–	–	B1 = 2, B2 = 1	B1 = 2	B1 = 1	–	B1 = 1	B1 = 1	–	–	B1 = 1

		O2 (n = 1)	O4 (n = 6)	O6 (n = 5)	O7 (n = 1)	O8 (n = 4)	O22 (n = 1)	O25 (n = 3)	O29 (n = 1)	O51 (n = 4)	O54 (n = 1)	O56 (n = 1)	O83 (n = 1)	O88 (n = 1)	O102 (n = 1)	O128 (n = 1)	Non (n = 3)
Cat	B1	–	–	–	1	1	–	–	1	–	–	–	–	1	1	–	–
	B2	1	6	5	–	–	1	3	–	4	1	1	1	–	–	1	1
	C	–	–	–	–	3	–	–	–	–	–	–	–	–	–	–	1
	D	–	–	–	–	–	–	–	–	–	–	–	–	–	–	–	1
	BPM	–	B2 = 2	B2 = 3	–	C = 1	–	–	B1 = 1	B2 = 1	–	–	–	B1 = 1	–	B2 = 1	B2 = 1, D = 1

*Non* non-typeable, *BPM* bacterial pneumonia-induced mortality.

## Discussion

Cases of ExPEC infections, including UTIs and meningitis, have been reported in various species. However, there is limited information on ExPEC-induced pneumonia. We previously reported suspected cases of ExPEC-induced pneumonia in dogs^16^. In addition, cases of *E. coli* infection in dog^20^ and cat^6^ lungs have also been reported, and the virulence factors associated with ExPEC are different from those linked to commensal or intestinal pathogenic *E. coli* strains^4^. Moreover, since *E. coli* is a primary infectious agent that decreases immunity to secondary infections by other pathogens^21^, the investigation of *E. coli* respiratory infections is imperative. To gain more insight into the characteristics of ExPEC-induced pneumonia in dogs and cats, the prevalence and pathogenicity of *E. coli* isolates from dog and cat lung samples were analyzed.

The present study described the characteristics of *E. coli* isolated from the lungs of dogs and cats that died due to bacterial pneumonia. The virulence factors *papA*, *hlyD*, and *cnf1* were relatively prevalent and exhibited differences in the frequency of their occurrence in pneumonia-induced mortality cases. A major virulence factor in ExPEC is α-hemolysin, which causes cell lysis and damage^12,22^. The *hlyCABD* operon codes for α-hemolysin, and *hlyD* participates in the secretion of α-hemolysin^23^. ExPEC infections are characterized by the presence of virulence factors like *cnf*, which causes apoptosis, and *papA*^24,25^. The *hly* virulence factor is present in the chromosomes of pathogenicity islands and is associated with other virulence factors, including *pap* and *cnf*^23,26^, indicating that these virulence factors are expressed together. Previous studies showed that ExPEC isolates from pneumonia-infected cats^6,13^ and dogs^15^ express *hly*, *cnf1*, and *papA*. In addition, lung injury was found to be severe in *hly*- and *cnf*-positive ExPEC strains in a rat pneumonia model^27^. The high frequency of occurrence of *papA*, *hlyD*, and *cnf1* in the present study suggests that *E. coli* strains, which possess these virulence factors, can cause bacterial pneumonia in dogs and cats, and may potentially lead to pneumonia-associated mortality.

The presence of *kps MII*, a virulence factor-encoding gene that also encodes for the K2 capsule protein, is a characteristic feature of neonatal meningitis caused by *E. coli*. It protects *E. coli* from phagocytosis and complement-mediated death^28,29^. In the present study, the *E. coli* isolates from neither species showed a significant association with the development of bacterial pneumonia, which may be attributed to *E. coli* viability. In the present study, *afa* was not detected in any *E. coli* isolates. This gene has been identified in uropathogenic *E. coli* (UPEC) and ExPEC strains but not in commensal strains of *E. coli*, which may indicate that it is not gastrointestinal-derived *E. coli*^30,31^. An earlier study showed that only the UTI isolates expressed *afa* from a total of 40 *E. coli* isolate samples collected from dogs and cats^32^, indicating that *afa* may not be highly prevalent in ExPEC strains. The adherence factors Foc and SfaS help the pathogen to bind to specific host receptors^33,34^. An earlier study showed that sfa-foc adhesion occurs in specific cell types, indicating differences in colonization depending on the host^35^. Therefore, in the present study, it is expected that the attachment of *E. coli* in the lung samples collected from dogs and cats was correlated with other adhesion molecules. The factor *iutA* engages in iron uptake, which is essential for colonization and bacterial growth in the host and becomes virulent in certain strains^36,37^. According to Landgraf et al.^37^, *iutA* is not essential for UPEC strains. In the present study, *iutA* was identified in some *E. coli* isolates from both species, and the frequency of occurrence of this gene was higher in the dog sample isolates than in the lung samples collected from cats. However, *iutA* did not correlate with bacterial pneumonia-induced mortality.

Shiga toxin 1 (stx1) and stx2 are the primary virulence factors of Shiga toxin-producing *E. coli* (STEC) strains, which cause diarrhea post-intestinal infection. In addition, intimin, a bacterial adhesion molecule that regulates intestinal epithelial cell adhesion of STEC strains, is encoded by *eae*^38^. The results from this study showed that *stx1*, *stx2*, and *eae* were not detected in the virulence factor analysis of *E. coli* isolates collected from the lung samples of dogs and cats. A previous study showed differences in *E. coli* isolates from fecal and lung samples^14^. This suggests that *E. coli* isolates from the lungs were derived from extraintestinal sources, and the possibility of contamination by intestinal *E. coli* after death or during autopsy^20^ can therefore be excluded.

In this study, bacterial pneumonia-induced mortality was higher in cats (34.3%) than in dogs (25.8%). Although more than 60% of *E. coli* isolates from cat lung samples contained *papA*, *hlyD*, and *cnf1*, no significant association between these virulence factors and mortality due to bacterial pneumonia, similar to the correlation established in the isolates from dog lung samples, could be established in cats. Most dogs used in this study were raised by humans as companion animals (55/66 cases), while more than half of the cats (19/35 cases) included in this study (data not shown) were strays. There is a possibility of differences in *E. coli* pathogenicity based on differences in the environment that these two species were exposed to. Earlier studies showed that *E. coli* strains infecting different species were similar, especially strains infecting humans and companion animals, and the possibility of inter-species *E. coli* transmission was suggested^39–41^. On the other hand, wild animals were infected with *E. coli* strains different from the strains infecting humans and other animals, and the infection was dependent on the species and its habitat^35,42^. Thus, the present study showed that the frequencies of occurrence of virulence factors in *E. coli* isolates from dogs and cats were different and were linked to differences in their habitats.

In this study, the phylogroups B1 (32.7%) and B2 (36.6%) were detected relatively more than the other phylogroups, and the mortality due to bacterial pneumonia was higher in these phylogroups (11/29 and 15/29 cases, respectively). The results showed that the virulence factors *papA*, *hlyD*, and *cnf1*, which are associated with bacterial pneumonia, were highly prevalent in phylogroup B2. Among the *E. coli* isolates from dogs and cats that died of bacterial pneumonia belonging to phylogroup B2, all seven isolates from dogs (7/7 cases) and seven out of eight isolates from cats (7/8 cases) were confirmed to contain these three virulence factors. *E. coli* isolates were mainly categorized into four phylogroups: A, B1, B2, and D. The commensal strains of *E. coli* mostly belong to phylogroups A and B1^1,4^, whereas the ExPEC strains belong to phylogroups B2 and D^3,14,15^. Previous studies showed that *E. coli* strains belonging to phylogroups B2 and D caused pathogenic symptoms due to the presence of various virulence factors^43^. In addition, most ExPEC strains, especially the strains causing UTIs, belong to phylogroups B2 and D and were characterized by the presence of virulence factors that may lead to extraintestinal infections^1,14,15,32^. Diseases caused by ExPEC strains expressing virulence factors, including *papA*, *hlyD*, and *cnf1*, in cats^44^, dogs^8^, and humans^45^ have been reported. The results from this study were consistent with those from earlier studies and established the correlation between *E. coli* strains belonging to phylogroup B2 that expressed virulence factors (*papA*, *hlyD*, and *cnf1*) and bacterial pneumonia-induced mortality.

Among the *E. coli* isolates from dog lung samples belonging to phylogroup B1, nine deaths were caused by bacterial pneumonia, but no correlation with the frequency of virulence factors could be established. Bacterial coinfections and secondary infections caused by *E. coli* are commonly associated with multiple pathogens, as well as primary infections^21,46^. Viral and bacterial coinfections have been commonly identified in humans^47^. An earlier study showed that the mortality rate increased as a result of severe lung lesions caused by coinfection by influenza and *E. coli* in a mouse model when compared to infections caused by a single pathogen^48^. The nature of *E. coli* isolates in the present study (primary or secondary infections) was not established. Nevertheless, it can be hypothesized that *E. coli* strains belonging to phylogroup B1 with a low frequency of occurrence of virulence factors may enhance pneumonia symptoms due to coinfection with other pathogens.

In this study, 22 and 15 O-serogroups were identified in the isolates from dog and cat lung samples, respectively. The O-antigen is an important virulence factor that is regulated by the repeating polysaccharide chain present in the outer membrane composed of lipopolysaccharides^46,49^. O-antigen subunits showed variability across different strains containing 181 O-antigens and promoted immune suppression associated with serum sensitivity, which is essential for pathogen survival and outbreak and epidemiological investigations^49–51^. In the present study, the O-serogroup patterns were different in the *E. coli* isolates from dog and cat lung samples; however, O4 and O6 serogroups were common in both species. Moreover, most isolates belonging to the serogroups O4 and O6 contained various virulence factors, including *papA*, *hlyD*, and *cnf1*. The results of the comparison between O-serogroup and virulence factors of two species showed *E. coli* isolates from lung samples of dogs belonging to serogroup O4 had significantly higher at bacterial pneumonia-induced mortality than other serogroups. In previous studies, various O-serogroups have been identified in ExPEC infections, and some O-serogroups were more prevalent in certain species. The serogroups O25, O2, O6, and O1 in patient samples were characterized by the presence of various virulence factors^51–53^. Isolated ExPEC samples from dogs and cats were grouped into serogroups O4 and O6 and characterized by the presence of various virulence factors^1,54^, specifically samples isolated from pneumonia-infected lungs of dogs^15,20^ and cats^6^ containing *papA*, *hlyD*, and *cnf1*. The results of comparison between O-serogroups and virulence factors from this study showed that serogroup O4 and O6 were relatively prevalent than other serogroups for virulence factors *papA*, *hlyD*, and *cnf1* with bacterial pneumonia in dogs and cats, although there was no significant association with bacterial pneumonia-induced mortality*,* except for serogroup O4 in dogs. Other O-serogroups have also been reported in addition to the dominant serogroups. According to Yuri et al.^54^, serogroups O25, O11, and O75 are commonly prevalent serogroups detected in canine UTIs. In human ExPEC cases, O18 associated with *sfaS* and *cnf1* expression in meningitis^1^, and O11, O17, and O77 associated with *papA*, *iutA*, and *kpsM II* expression^55^ have been reported. In the present study, in addition to the dominant O4 and O6 serogroups, other O-serogroups were also identified, which may be associated with ExPEC pathogenicity. The relationship analysis between phylogroups and O-serogroups showed that most isolates belonged to serogroups O4 and were categorized under phylogroup B2. According to previous studies, pathogenic O-serogroups are associated with phylogroup B2 that is characterized by the presence of various virulence factors^56,57^. Serogroups O4 and O6 were not correlated with bacterial pneumonia since *E. coli* isolates were not concentrated in specific O-serogroups and various O-serogroups were identified. Most isolates belonging to serogroup O4 and O6 were classified under phylogroup B2 that is associated with bacterial pneumonia, suggesting a possible correlation between bacterial pneumonia and frequency of occurrence of virulence factors.

In conclusion, this study showed that ExPEC can cause pneumonia in dogs and cats and is an important pathogen implicated in the death of animals depending on pathogenicity. Pathogenicity analysis confirmed the correlation between frequency of occurrence of virulence factors and bacterial pneumonia-induced mortality, and this association was observed in specific ExPEC strains that contained the virulence factor-encoding genes *papA*, *hlyD*, and *cnf1*. In addition, bacterial pneumonia-induced mortality in dogs is more likely to be associated with strains belonging to phylogroups B2 and O-serogroups O4 and O6, and although there were no significant differences in association, strains belonging to phylogroups B2 and O-serogroups O4 and O6 were also dominant in cats which died from bacterial pneumonia. The results of this study described the prevalence and pathogenicity of ExPEC in lung samples collected from dogs and cats in South Korea and provide insights into the correlation between ExPEC strains and bacterial pneumonia.

## Materials and methods

### *E. coli* isolates

From January 2020 to June 2022, 101 lung samples from 66 dogs and 35 cats (companion and stray dogs and cats) that died in animal hospitals and outdoors were collected. The sample collection date and location were recorded using application documents. All lung samples were submitted to the Animal and Plant Quarantine Agency (APQA, South Korea) for diagnosis of gross and histopathological lesions. Following the APQA standard autopsy guidelines, samples with bacterial lesions of the lungs, including fibrinous and suppurative pneumonia, pleurisy, and bacterial colonies were identified but no lesions were identified in other organs and were declared death due to bacterial pneumonia. After the autopsy, all samples were stored at 4 ℃ until further experimentation. Samples were first streaked onto MacConkey (MAC; BD, Franklin Lakes, NJ, USA) and sheep blood agar (BA; Asan, Korea), and then incubated in an atmosphere of 5% CO_2_ at 37 ℃ for 15–18 h. Pure colonies were transferred to a new BA plate and incubated under similar conditions for the same duration. Pure colonies were then isolated, and bacterial species were identified using the VITEK 2 system (bioMérieux, Craponne, France). All *E. coli* isolates were stored using the BRIX Microvials system (BASIC SCIENCE, Korea) at − 80 ℃ until further experimentation.

### Determination of occurrence frequency of virulence factors and phylogenetic analysis

Genomic DNA was extracted using the boiling method. Specific primers for the virulence factors used in this study are listed in Table 1. Amplification was performed in a thermocycler (Takara, Shiga, Japan) with specific cycling conditions. The cycling conditions for *focG*, *kps MII*, *papA*, *sfaS*, *afa*, *hlyD*, and *iutA* were as follows: an initial cycle at 95 ℃ for 15 min, 35 amplification cycles consisting of denaturation at 95 ℃ for 30 s, annealing at 60 ℃ for 30 s, followed by extension at 72 ℃ for 30 s, and a final cycle of extension at 72 ℃ for 10 min^17^. The cycling conditions for *stx1*, *stx2*, *eae*, and *cnf1* are as follows: an initial cycle at 94 ℃ for 5 min, 35 to 40 amplification cycles consisting of denaturation at 94 ℃ for 1 min, annealing at 55 ℃ for 1 min, followed by extension at 72 ℃ for 1 min, and a final cycle of extension at 72 ℃ for 10 min^18,19^. Phylogenetic analysis of the 101 *E. coli* isolates was performed based on PCR analysis of *chuA*, *yjaA*, *arpA*, *TspE4.C2*, and *trpA*, according to the previously established procedure^58^. Based on the PCR results, each isolate was assigned to a specific phylogenetic group (A, B1, B2, C, D, E, and F).

### O-serogrouping

O-serogroups were determined using PCR, according to the previously described procedure^59^. *E. coli* isolates were cultured on BA at 37 ℃ overnight. Genomic DNA was extracted by boiling the cultured colonies. O-genotyping multiplex PCR was performed using primer sets MP-1–MP-20.

### Statistical analysis

All results are expressed as percentage of isolates and presence of virulence factors. The frequency of occurrence of virulence factors in *E. coli* isolates from dog and cat lung samples was statistically compared using the chi-squared test or Fisher’s exact test followed by Holm’s *post-hoc* test. The *P*-value was calculated, and statistical significance was set at *P* < 0.05.

### Ethics approval and consent to participate

Informed consent was obtained from all dog and cat owners involved in the study.

## Data Availability

The data presented in this study are available on request from the corresponding author.
